# Evolution of DC-SIGN use revealed by fitness studies of R5 HIV-1 variants emerging during AIDS progression

**DOI:** 10.1186/1742-4690-5-28

**Published:** 2008-03-27

**Authors:** Marie Borggren, Johanna Repits, Carlotta Kuylenstierna, Jasminka Sterjovski, Melissa J Churchill, Damian FJ Purcell, Anders Karlsson, Jan Albert, Paul R Gorry, Marianne Jansson

**Affiliations:** 1 Dept Laboratory Medicine, Lund University, Lund, Sweden; 2 Center for Infectious Medicine, Karolinska Institute, Stockholm, Sweden; 3 Macfarlane Burnet Institute for Medical Research and Public Health, Melbourne, Australia; 4 Department of Medicine, Monash University, Melbourne, Australia; 5 Department of Microbiology and Immunology, University of Melbourne, Australia; 6 Venhälsan (Gay Men's Health Clinic), Karolinska Institute, Department of Clinical Science and Education, Södersjukhuset, Stockholm, Sweden; 7 Dept of Microbiology, Tumor and Cell biology (MTC), Karolinska Institute, Stockholm, Sweden; 8 Dept of Virology, Swedish Institute for Infectious Disease Control (SMI), Solna, Sweden

## Abstract

**Background:**

At early stages of infection CCR5 is the predominant HIV-1 coreceptor, but in approximately 50% of those infected CXCR4-using viruses emerge with disease progression. This coreceptor switch is correlated with an accelerated progression. However, those that maintain virus exclusively restricted to CCR5 (R5) also develop AIDS. We have previously reported that R5 variants in these "non-switch virus" patients evolve during disease progression towards a more replicative phenotype exhibiting altered CCR5 coreceptor interactions. DC-SIGN is a C-type lectin expressed by dendritic cells that HIV-1 may bind and utilize for enhanced infection of T cells in *trans*. To further explore the evolution of the R5 phenotype we analyzed sequential R5 isolates obtained before and after AIDS onset, i.e. at the chronic stage and during end-stage disease, with regard to efficiency of DC-SIGN use in *trans*-infections.

**Results:**

Results from binding and *trans*-infection assays showed that R5 viruses emerging during end-stage AIDS disease displayed reduced ability to use DC-SIGN. To better understand viral determinants underlying altered DC-SIGN usage by R5 viruses, we cloned and sequenced the HIV-1 *env *gene. We found that end-stage R5 viruses lacked potential N-linked glycosylation sites (PNGS) in the gp120 V2 and V4 regions, which were present in the majority of the chronic stage R5 variants. One of these sites, amino acid position 160 (aa160) in the V2 region, also correlated with efficient use of DC-SIGN for binding and *trans*-infections. In fitness assays, where head-to-head competitions between chronic stage and AIDS R5 viruses were setup in parallel direct and DC-SIGN-mediated infections, results were further supported. Competitions revealed that R5 viruses obtained before AIDS onset, containing the V2 PNGS at aa160, were selected for in the *trans*-infection. Whereas, in agreement with our previous studies, the opposite was seen in direct target cell infections where end-stage viruses out-competed the chronic stage viruses.

**Conclusion:**

Results of our study suggest R5 virus variants with diverse fitness for direct and DC-SIGN-mediated *trans*-infections evolve within infected individuals at end-stage disease. In addition, our results point to the importance of a glycosylation site within the gp120 V2 region for efficient DC-SIGN use of HIV-1 R5 viruses.

## Background

Human immunodeficiency virus type 1 (HIV-1) infection requires the expression of CD4 in addition to a coreceptor, either CCR5 or CXCR4, on the surface of the target cell. Evolution of the HIV-1 phenotype during disease progression has primarily been related to coreceptor usage where early during infection viruses primarily use CCR5 (the R5 phenotype) [1], and later during disease progression, viruses with the ability to use CXCR4 emerge (the X4 phenotype) [2,3]. The appearance of X4 virus, which can be seen in approximately 50% of those infected, is associated with accelerating loss of CD4 cell and more aggressive disease progression [2,4,5]. However, the remaining half of the patients that maintain exclusively CCR5 restricted (R5) viruses still progress to AIDS eventually. Nevertheless, the R5 phenotype of viruses from these "non-switch virus" patients have in studies by us and others been shown to evolve with disease progression in properties such as replicative capacity, cytopathicity, fusogenicity, sensitivity to chemokines and other entry inhibitors, in addition to mode of coreceptor use [6-13].

The main target cells for HIV-1 are CD4+ T cells, but macrophages and dendritic cells (DCs) are also infected. Even though DCs are susceptible to HIV-1 infection [14,15], HIV-1 does not appear to be efficiently produced by these cells [16,17]. A more important role of DCs in HIV-1 pathogenesis may instead be the ability of the virus to use DCs for efficient *trans*-infection of T cells. C-type lectins, such as DC-SIGN (dendritic cell specific ICAM3-grabbing non-integrin), expressed on the surface of DC and macrophages has been implicated to play a key role in these *trans*-infections [18-21]. DC-SIGN has been shown to enhance HIV-1 *in vitro *infections in both *trans*- and *cis*-fashion [18,22], but *in vivo *DC-SIGN might be one of many options for DC to transfer virus to T cells [23,24]. HIV-1 binds to DC-SIGN through the outer envelope glycoprotein gp120 [18], but exactly how this interaction occur is still not clear. Several studies have indicated that mannose residues on N-linked glycans of gp120 are required for DC-SIGN binding [25-28]. However, if it is single glycans or combination of many such residues in gp120 that are responsible for DC-SIGN binding remains unclear. Nevertheless, N-linked glycosylation sites within gp120 have been implicated in enhanced binding of DC-SIGN in SHIV transmission [28]. In addition, DC-SIGN binding has recently been reported to overlap with N-linked glycans within the epitope recognized by the 2G12 monoclonal antibody [26].

Importance of DC-SIGN use at the event of viral transmission has been suggested [24,29], however, little is known on the evolution of DC-SIGN use within infected individuals along with disease progression. However it was recently reported that HIV-1 DC-SIGN use varied according to coreceptor use within a single infected patient [30]. In the present study we have characterized a panel of R5 HIV-1 isolates obtained sequentially from non-switch virus patients before and after AIDS onset with regard to DC-SIGN use. The R5 isolates were tested in binding, *trans*-infection and competition assays and results revealed that DC-SIGN use of end-stage AIDS isolates were impaired as compared to their corresponding chronic phase R5 viruses. In this study we also set out to identify viral determinants that could be correlated to efficient DC-SIGN use. We found a potential N-linked glycosylation site (PNGS) in the V2 region of gp120 that correlated with enhanced binding and use of DC-SIGN in *trans*-infections.

## Results

### DC-SIGN binding and trans-infection efficacy evaluated for sequentially obtained chronic and end-stage R5 HIV-1 isolates

**Figure 1:**
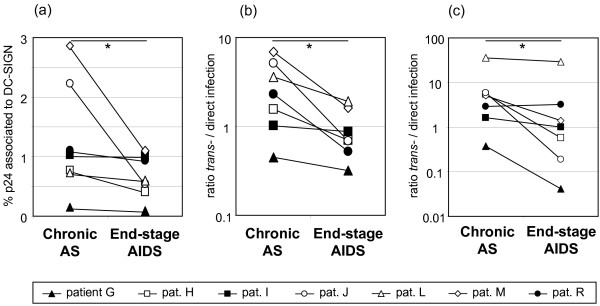
**Ability of sequential R5 isolates to bind and utilize DC-SIGN for *trans*-infection**. (A) DC-SIGN binding ability assessed as percentage specifically DC-SIGN associated p24 antigen. Efficiency of DC-SIGN mediated *trans*-infections analysed as ratios of p24 antigen release in DC-SIGN mediated PBMC (B) or T-cell line C6 (C) infections over p24 antigen release in directly infected PBMC and C6 cultures respectively. Presented data are average from results obtained in two or three assays performed. *, p < 0.05

We recently reported on the phenotypic evolution of R5 isolates during disease progression in patients who retain CCR5-dependent HIV-1 isolates throughout the entire disease course [10,12]. To analyse if DC-SIGN use also may evolve, sequential R5 isolates obtained before and after AIDS onset from seven patients were examined for ability to bind DC-SIGN. Virus was in parallel added to wild type (wt) and DC-SIGN expressing Ramos B-cells, and percentage specifically DC-SIGN bound viral p24 antigen was analysed. We found that AIDS R5 isolates from six out of the seven patients showed reduced ability to bind DC-SIGN (Fig. 1a), (p = 0.018). Next, since we previously noted that chronic and end-stage R5 viruses display diverse infectivity [12], we tested the same set of R5 isolates for relative efficacy of DC-SIGN mediated trans-infection. Direct target cell infections in PBMC and a CCR5-expressing T-cell line (C6), were set up in parallel with DC-SIGN mediated trans-infections, i.e. cocultures of target cells and Ramos/DC-SIGN cells or Ramos/wt cells that had been pre-pulsed with virus. The relative efficacy of DC-SIGN use was then assessed as ratios of p24 antigen in DC-SIGN mediated infections over p24 in directly infected cultures. As expected, control *trans*-infections with Ramos/wt showed no infection of target cells (data not shown). Instead, in DC-SIGN-mediated *trans*-infections we found that all AIDS R5 isolates used DC-SIGN for *trans*-infection of PBMC less efficiently than the corresponding R5 isolates obtained during the chronic phase, before AIDS onset (Fig. 1b), (p = 0.018). Similarly, DC-SIGN-mediated *trans*-infection of the T cell line was reduced in six out of seven end-stage R5 virus cultures (Fig 1c), (p = 0.028). Thus, our results suggest that HIV-1 R5 variants with reduced ability to bind and use DC-SIGN for efficient trans-infection may emerge after AIDS development.

### End-stage R5 viruses display loss of PNGS in gp120 V2 and V4 regions

**Figure 2:**
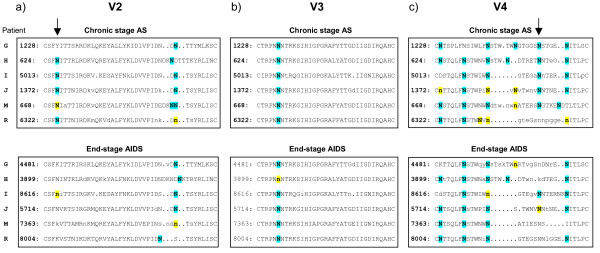
**Gp120 V2, V3 and V4 loop region amino acid sequences of chronic and end-stage R5 sequences**. Each sequence depicts the consensus V2 (a), V3 (b) and V4 (c) loop sequences of four clones obtained from chronic asymptomatic (AS) and end-stage AIDS R5 isolates. Upper case letters illustrate amino acids present in all analyzed clones, i.e homogenous sites, lower case letters represent dominating amino acids in polymorphic sites. Bold letters show potential N-linked glycosylation sites (PNGS), blue shaded sites display homogenous PNGS and yellow shaded sites represent polymorphic PNGS. The arrows point out the V2 aa160 and the V4 aa406 positions.

With the aim to identify viral determinants that could account for the observed differences in DC-SIGN use of R5 viruses isolated during end-stage disease progression, we next set out to analyze the glycosylation pattern within the outer envelope glycoprotein gp120. For this purpose, the *env *gene was amplified and cloned from R5 isolates obtained sequentially before and after AIDS onset from six of the patients. For each isolate the *env *gene of four clones was sequenced [GenBank: EF600067–EF600114] and by the use of the N-glycosite tool [31] potential N-linked glycosylation sites (PNGS) in gp120 were identified. This analysis revealed two PNGS within gp120, aa160 in the V2 region and aa406 in the V4 region (according to reference strain HXB2 [32]), that discriminated R5 viruses obtained before and after AIDS onset (Fig. 2). These two PNGS were significantly more frequent in the chronic R5 isolates as compared to the viruses obtained at end-stage disease. In V2 aa160 18 out of 24 of the *env *sequences obtained prior to AIDS onset had a PNGS while only four out of 24 *env *sequences obtained after AIDS diagnosis displayed this glycosylation site (p < 0.001). Likewise, in the V4 aa406 site 20 out of 24 *env *sequences from the chronic phase had PNGS but only six out of 24 end-stage sequences had the site (p < 0.001). Since modifications, including both PNGS and charge, in the V3 region has been reported to affect DC-SIGN binding and subsequent transfer [30], we also compared V3 loop sequences of R5 virus obtained at chronic and end-stage disease. However, in contrast to the V2 and V4 regions the V3 loop sequences were highly conserved and could not segregate R5 virus obtained before and after AIDS onset (Fig. 2). Thus, consensus sequences generated from four *env *clones of each R5 isolate showed that a majority of the chronic phase viruses had PNGS in V2 aa160 and V4 aa406, while these sites were rarely found among R5 viruses isolated after AIDS onset (Figure 2). Therefore, we conclude that R5 virus variants that lack glycosylation sites in the gp120 V2 and V4 regions may emerge at end-stage disease.

### PNGS in the N-terminus of the gp120 V2 region relates to efficiency of DC-SIGN use

**Figure 3:**
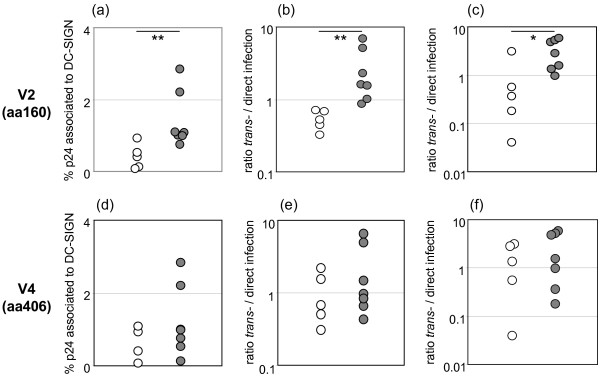
**DC-SIGN use of R5 isolates in relation to PNGS in gp120 V2 (aa160) and V4 (aa406) regions**. Figures display results from (a, d) binding assays, (b, e), trans-infections of PBMC and (c, f) trans-infections of C6 cells as target cells. Isolates were classified according to presence or absence of PNGS; open circles, isolates completely lacking PNGS; dark circles, isolates displaying PNGS in at least one of the clones sequenced. Presented data are the averages from results obtained in two or three assays performed. *p < 0.05; ** p < 0.01.

We next analyzed if efficient DC-SIGN use correlated with presence or absence of the V2 aa160 or V4 aa406 PNGS. Separately for the V2 and V4 PNGS the R5 isolates were for this purpose divided into groups, either harboring PNGS in any of the four *env *sequences of each isolate or completely lacking the site. Efficiency of DC-SIGN use, i.e. % specific binding and p24 antigen ratios from *trans*-over direct infections, were then compared between the groups (Fig. 3). Results showed that R5 isolates harboring the V2 glycosylation site bound DC-SIGN clearly better than R5 isolates lacking this site (Figure 3a), (p = 0.005). Furthermore, the same pattern was seen in relation to *trans*-infections of PBMC and the T-cell line, where R5 isolates with the V2 glycosylation site benefited significantly more from DC-SIGN-meditated *trans*-infections as compared to isolates completely lacking the site (Figure 3b and 3c) (p = 0.002 and p = 0.03). However, at the analysis of the V4 PNGS in relation to DC-SIGN binding and utilization, no significant correlations were noted (Figure 3d–f). Moreover, DC-SIGN binding and trans-infection efficiency did not differ when R5 isolates harboring the V2 aa160 or V4 aa406 PNGS in all four env sequences were compared with those isolates being polymorphic for these sites (data not shown). To summarize, results suggest that a PNGS in the N-terminus of the gp120 V2 region, aa160, of R5 viruses contributes to efficient DC-SIGN binding and *trans*-infections of target T-cells.

### Diverse fitness of chronic and end-stage R5 variants, with and without V2 PNGS respectively, in DC-SIGN mediated and direct infections

**Figure 4:**
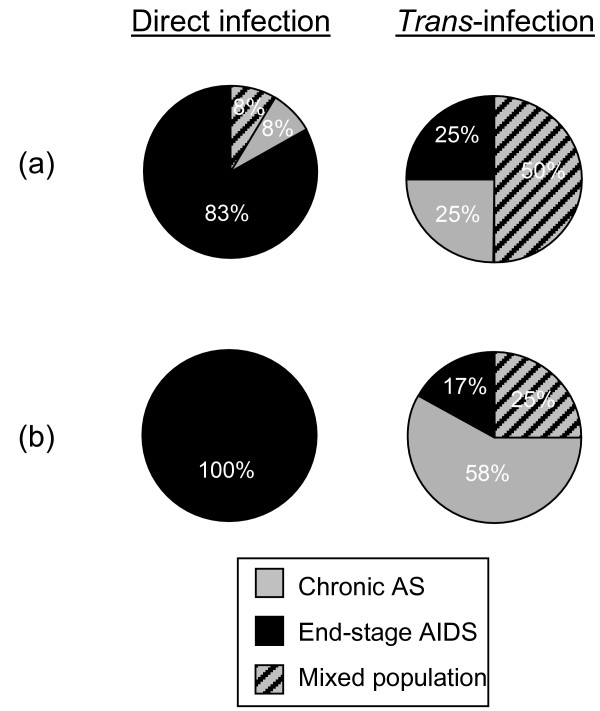
***In vitro *selection of R5 HIV variants in head-to-head competitions in direct and DC-SIGN-mediated infections**. Competition assays were set-up with **(a) **inter-patient mixed chronic phase and end-stage R5 isolates from patients R and G, respectively and with **(b) **intra-patient mixed chronic AS and end-stage AIDS R5 isolates from patient J. Replicating viruses were identified by V2 region sequencing and presented percentage were calculated from ten to twelve parallel infections.

To further investigate the impact of the gp120 V2 PNGS in aa160 of HIV-1 R5 viruses for efficient DC-SIGN use we set up competition assays. On the basis of our previous findings showing that R5 isolates obtained before and after AIDS onset display varying ability to infect target cells in a direct manner [12], divergent utilization of DC-SIGN for *trans*-infections (Fig. 1) and differences in the V2 PNGS aa160 (Figure 2), we chose to set up head-to-head competition assays to test relative fitness of the viruses in direct and DC-SIGN mediated infections. Thus, R5 viruses were mixed in equal concentrations, serially diluted and used for parallel set-up of direct and DC-SIGN-mediated PBMC infections. Replicating virus variants were identified by sequencing of the gp120 V2 region, known to harbour isolate specific variations (Fig. 2). Initially competitions were set up between interpatient R5 viruses using chronic and end-stage isolates from patients R and G, respectively (Figure 4a), since these isolates fulfilled the criteria being homozygous for the V2 PNGS aa160 or completely lacking the V2 PNGS aa160, respectively. Next intrapatient virus competitions were set up with chronic and end-stage R5 isolates from patient J (Figure 4b), since also these isolates were homozygous for V2 PNGS aa160 or lacked this site, respectively. Results revealed that in the DC-SIGN-mediated *trans*-infections the chronic stage R5 viruses out-competed the end-stage AIDS viruses in both interpatient and intrapatient virus competitions, while the opposite was seen in the direct PBMC infections (Figure 4) (p < 0.021 and p < 0.0022 respectively). Thus, end-stage R5 viruses dominated over chronic stage viruses in the direct PBMC infections, 83 and 100% positive cultures in the inter-and intrapatient competitions respectively. In contrast, in the DC-SIGN-mediated *trans*-infections only 25 and 17% of the cultures were dominated by R5 virus from the end-stage AIDS phase. R5 variants obtained during the asymptomatic chronic phase were instead clearly detected in a majority of DC-SIGN-mediated infections, either exclusively or mixed with the end-stage variants. Thus, results from the competition assays revealed *in vitro *selection of chronic stage R5 viruses, harbouring V2 PNGS aa160, in the DC-SIGN-mediated *trans*-infections, while end-stage R5 variants lacking V2 PNGS aa160 dominated in the direct PBMC infections.

## Discussion

By the use of binding, *trans*-infection and head-to-head competition assays we have in this study revealed that DC-SIGN use of R5 HIV-1 variants evolves within single individuals along disease progression. R5 virus that utilizes DC-SIGN less efficiently may emerge at end-stage disease. This study also shows that efficient use of DC-SIGN correlates with the presence of a specific potential N-linked glycosylation site in the gp120 V2 region.

Differences in DC-SIGN use were reported in previous studies that compared unrelated HIV-1 isolates [19] or CCR5- and CXCR4-using viruses obtained from one patient [30]. The present study confirms that the ability to use DC-SIGN may vary between different HIV-1 variants. In addition, our study demonstrates that the ability of HIV-1 R5 viruses to bind and use DC-SIGN for trans-infection may evolve within single infected individuals. Since the chronic and end-stage R5 isolates that we studied displayed diverse replicative capacity in PBMC [12], we took care to study the relative efficacy of DC-SIGN utilization by setting up parallel direct target cell infections and DC-SIGN mediated infections. We found that R5 isolates with reduced ability to utilize DC-SIGN for trans-infection may emerge after AIDS onset. This observation supports our earlier findings on viral phenotypic evolution along with disease progression in patients who retain CCR5 restricted HIV-1 isolates until end-stage disease [6,7,10,12]. Additional evidence for this was provided by the results from the head-to-head competition assays where end-stage R5 viruses displayed enhanced fitness in the direct PBMC infections compared to chronic R5 viruses. Interestingly, the opposite outcome was observed in the DC-SIGN mediated competitions, where the chronic R5 isolates displayed superior fitness. Thus, R5 variants emerging after AIDS onset appear more fit in direct target cell infections, while they benefit less from DC-SIGN mediated trans-infections.

In our attempts to identify viral determinants for efficient DC-SIGN use related to glycosylation patterns of the envelope glycoproteins we cloned and sequenced the *env *gene of R5 viruses isolated sequentially during the chronic asymptomatic phase, and at the end-stage AIDS phase. Here we found that PNGS in two specific gp120 locations, aa160 in the N-terminus of the V2 region and aa406 in the C-terminus of the V4 region, differed over time in *env *sequences of the six patients. In contrast, the V3 loop sequences were highly conserved comparing the sequential viruses and could not discriminate between R5 viruses obtained before and after AIDS onset, suggesting that alterations in this region may not be accepted by CCR5 restricted HIV-1 variants in patients that maintain viral populations being exclusively of R5 phenotype during the whole disease course. Next, when comparing results from binding and *trans*-infection assays with the presence or absence of PNGS in these two locations we observed that the V2 region, aa160, PNGS correlated with the efficiency of these viruses to bind and use DC-SIGN for trans-infections. Also results from the competition assays supported the observation, since chronic stage R5 viruses with a PNGS in the V2 aa160 site dominated in the DC-SIGN mediated trans-infections, despite the fact that the end-stage viruses out competed the chronic stage viruses in the direct target cell infections. The importance of the V2 aa160 PNGS in DC-SIGN-mediated trans-infections is also strengthened by the fact that both the intrapatient competition, where the viral backbones of chronic and end-stage R5 viruses are of similar origin, and the interpatient competition, where the viral backbones are less related since they have developed in separate hosts, revealed the same results. In contrast, no statistical correlations between PNGS in the V4 aa406 and DC-SIGN use were found. These data are in agreement with those of Lue and colleagues, who reported that the presence of a PNGS in the gp120 aa160 position within the V2 loop of SHIV SF162 correlated with increased binding to DC-SIGN [28]. In was also reported that enhanced DC-SIGN binding correlated with increased mucosal transmission of SHIV SF162P3, which was the virus variant that harboured PNGS aa160 in contrast to the less transmissible parental virus strain SHIV SF162 that lacked the site. These observations, taken together with the results of the present study suggest that the aa160 PNGS in the N-terminus of the gp120 V2 region contributes to the binding of R5 envelope glycoproteins to DC-SIGN.

The glycosylation site in position aa160 of gp120 V2 region has in HxB2 [33] and SF2 [32] been reported to be of complex carbohydrate type. However, initial data suggested that high mannose oligosaccharides on gp120 are responsible for binding to DC-SIGN [34]. Thus, the contribution of the aa160 PNGS for DC-SIGN use that we here report stands in contradiction to this and other studies claiming solely high mannose glycans as responsible for DC-SIGN interactions [25,26]. Instead, recent studies have revealed a wider range of glycan ligands for DC-SIGN including Lewis X antigen [35] and complex-type N-glycans [36]. An alternative explanation for our observation could be that multiple glycans associated with gp120 are involved in DC-SIGN binding, including both high-mannose and complex type, where loss or blockade of single such residues results in decreased binding affinity. Another reason for different results could be structural interactions between the glycan in aa160 and high mannose glycans in gp120, where aa160 glycan might not be the actual binding site for DC-SIGN but an important modifier of the gp120 structure necessary for DC-SIGN binding. In fact, a great majority of all HIV-1 sequences reported in the Los Alamos HIV Sequence Database [31] harbour the V2 aa160 PNGS, which may explain why most HIV-1 variants so far tested may utilize DC-SIGN in a relative efficient manner [18,19,22,24]. It might also be that glycans by themselves are not enough for optimal DC-SIGN use, since it recently was reported that multiple modifications of gp120, including V1 and V2 length and V3 charge, in combination with the N-linked glycosylation pattern affected DC-SIGN use [30]. Nevertheless, the impact of structural determinants within gp120 for optimal use of C-type lectins, including DC-SIGN, merits further studies since such alterations has been shown to not only impact the HIV *trans*-infection but also play a role in the immunoregulatory effects mediated by the virus [37].

Findings such as the preferential binding of virus by DC-SIGN expressing cells of rectal mucosa [38] and the accumulation of DC-SIGN expressing DCs in lymphoid tissue following acute HIV infection [39], suggest that virus DC-SIGN interactions may play a critical role in the early events of an HIV infection. Enhanced mucosal transmissibility of viruses with efficient gp120-DC-SIGN binding was also suggested comparing different SHIV strains [28]. However, it has not been ruled out if the infection enhancement effects mediated by DC-SIGN are in fact *cis *or *trans *effects, since it appears to be two phases of DC-mediated HIV transfer to CD4+ T cells involving both of these mechanisms [21]. Still, the question is open as to the importance of DC-SIGN for virus transmission *in vivo *[24,40] since other DC expressed C-type lectins might be of equal importance [24,41]. However, studies on DC-SIGN mediated *trans*-infection may add to the understanding of viral interaction between HIV-1 and a wider range of DC expressed C-type lectins, since these receptors recognize carbohydrate domains on the viral envelope [42].

In addition, this study which focuses on DC-SIGN use of R5 viruses sequentially isolated during disease progression may shed light on virus evolution during end-stage disease progression. R5 virus variants emerging late in the disease appear to be dispensable with respect to efficient DC-SIGN use. Instead, after AIDS onset during severe immunodeficiency, changes in the viral envelope may favour virus variants with increased affinity for the specific receptors used in direct target cell infections. Reason for this loss of efficient DC-SIGN use at end-stage disease seems to be a naturally occurring change in the glycosylation pattern of the HIV-1 envelope glycoprotein gp120. Thus, we speculate that virus DC-SIGN interactions are of greater importance in the earlier phases of the HIV-1 pathogenesis. The sustained efficient DC-SIGN use, from primary infection to the chronic stage, may be the result of virus immune evasion from neutralizing antibodies. Indeed, it has recently been suggested that DC-SIGN-mediated capture of neutralized HIV-1 by dendritic cells may result in immune evasion from the neutralizing effects of potentially neutralizing antibodies [43]. In another study, on SIV interactions with DC-SIGN, it has also been reported that virus binding to DC-SIGN conferred neutralization resistance to an otherwise sensitive SIV variant [44]. Thus, during the chronic and relatively immunocompetent phase of the infection efficient DC-SIGN use could be an important viral feature selected for, while at severe immunodeficiency, during end-stage disease, the lack of proper antibody responses may result in the emergence of virus variants that instead display enhanced fitness for direct target cell infection. To determine the relative efficacy of DC-SIGN use it would be of interest to compare viruses sequentially obtained during the complete disease course, from the time point of primary infection to the chronic and end-stage disease phases. Such studies together with the results presented here may add knowledge on evolution of the HIV-1 phenotype at different stages of the infection which in turn may help in rational vaccine design and development of therapeutics.

## Conclusion

This study shows that the ability of R5 HIV-1 to bind and utilize DC-SIGN may vary, both between patients and over time. By the comparison of R5 viruses obtained sequentially during disease progression we found that end-stage R5 isolates obtained after AIDS onset display reduced ability to utilize DC-SIGN as compared to isolates obtained earlier during the chronic but asymptomatic phase. In agreement with this observation, head-to-head competition assays revealed that chronic R5 viruses were selected for in DC-SIGN mediated trans-infections, while the opposite was noted in the direct PBMC infections where end-stage R5 isolates dominated. In addition, results suggest that PNGS within the gp120 V2 region contributes to efficient DC-SIGN use since chronic stage R5 viruses harbouring this site display enhanced DC-SIGN binding and use, and were also selected for in the *trans*-infection competition assays. These results suggest that R5 HIV-1 variants with diverse fitness for direct and DC-SIGN mediated infections may emerge with disease progression. Also, efficient utilization of DC-SIGN by R5 HIV-1 seems less important after AIDS onset. Furthermore, the loss of a glycosylation site within the gp120 V2 region in end-stage R5 viruses may contribute to the observed reduction in the use of DC-SIGN.

## Materials and methods

### Virus isolates

Primary HIV-1 isolates were sequentially obtained from patients within a cohort of homo- and bi-sexual men attending a STI/HIV clinic in Stockholm, Sweden. The selected patients maintained CCR5-restricted (R5) isolates during the entire course of the disease, i.e. during the asymptomatic chronic phase and during the end-stage AIDS phase, table 1. Patient clinical statuses and virus biological phenotypes were previously described [2,6,7,10,12]. Virus stocks were produced by propagation in PHA stimulated peripheral blood mononuclear cells, PBMC, from healthy donors.

**Table 1:** Patient clinical status, CD4 count at time of virus isolation, time to/from AIDS diagnosis and coreceptor use of primary isolates studied.

**Patient**^a^	**Isolate**	**CD4 count**^b^	**Months to AIDS**^c^	**Clinical status**^d^	**Coreceptor use**^e^
G	1228	260	-9	Chronic AS	R5
	4481	5	+26	End-stage AIDS	R5
H	624	290	-27	Chronic AS	R5
	3899	6	+6	End-stage AIDS	R5
I	5013	140	-30	Chronic AS	R5
	8616	90	+11	End-stage AIDS	R5
J	1372	220	-11	Chronic AS	R5
	5714	20	+20	End-stage AIDS	R5
L	462	220	-44	Chronic AS	R5
	3932	13	+/-0	End-stage AIDS	R5
M	668	750	-54	Chronic AS	R5
	7363	20	+20	End-stage AIDS	R5
R	6322	200	-2	Chronic AS	R3R5
	8004	9	+16	End-stage AIDS	R3R5

a) Patient code according to [7].

b) CD4^+ ^T cells/μl at time of virus isolation.

c) Time point of virus isolation related to months before and after AIDS diagnosis.

d) Patient status at time of virus isolation. AS = asymptomatic or with mild symptoms not classified as AIDS, AIDS = acquired immunodeficiency syndrome.

e) Coreceptor use determined by infection of U87.CD4 and GHOST coreceptor indicator cell lines expressing CCR2b, CCR3, CCR5, CXCR4, CXCR6 or BOB [7].

### Cells

PBMC from healthy donors were activated for 2–3 days in complete medium, i.e. RPMI 1640 with 10% FCS and antibiotics, supplemented with 2 μg/ml phytohaemagglutinin (PHA). Ramos cells, wild-type (wt) and DC-SIGN expressing [45], were maintained in IMDM medium with 10% FCS and antibiotics. The 'C6' cell line, which is based on CEM174 cells and engineered to express CCR5 as described [46], was kindly provided by Dr David Dorsky, University of Connecticut Health Center, USA. C6 cells were maintained in RPMI 1640 medium with 5% FCS and antibiotics.

### Generation of full length *env *clones and Gp160 sequencing

Genomic DNA was extracted from PBMC infected with the HIV-1 R5 isolates seven days post infection, using a DNeasy DNA extraction kit (Qiagen) according to the manufacturer's protocol. HIV-1 *env *genes were amplified from genomic DNA using Expand high fidelity DNA polymerase and nested PCR approach as described previously [13,47,48]. The outer primers were Env1A and Env1M [49] and the inner primers were Env-KpnI and Env-BamHI [50] which amplifies a 2.1 kb fragment of HIV-1 *env *corresponding to nucleotides 6,348 to 8,478 of HxB2 and spans unique KpnI and BamHI restriction sites. PCR was performed with an initial denaturation step of 94°C for 2 min, followed by 29 cycles of 95°C for 15 s, 60°C for 30 s, and 72°C for 2 min, and a final extension of 72°C for 7 min. During the last 20 cycles the extension time was increased by an additional 5 s per cycle. PCR product DNA was purified over a column using High Pure PCR Product Purification Kit (Roche) and cloned into the pSVIIIenv expression plasmid [48,49] by replacement of the 2.1 kb KpnI to BamHI HxB2 *env *fragment. Thus, the cloned e*nv *fragments contain the entire gp160 coding region except for 36 amino acids at the N-terminus and 105 amino acids at the C-terminus, which in the pSVIII plasmid derived from HxB2.

Plasmid DNA was used as template for gp160 sequence analysis of the *env *clones and from each R5 isolate four clones were selected according to functionality in a single round entry assay described previously [13,47,51]. A set of 7 forward; F1EnvJR (5'-G/CAGAAAGAG-CAGAAGACAGTGGCAATGA-3'), F2EnvJR (5'-GTCTATTATGGGGTACCTGTGTGG-3'), F3EnvJR (5'-GTGTACCCACAGACCCCAACCCACAAG-3'), F4EnvJR (5'-ACAA-TGC/TACACATGGAATTAA/GGCCA-3'). F5EnvJR (5'-TTTAATTGTGGAGGGGAAT-TTTTCT-3'), F6EnvJR (5'-GTGGGAATAGGAGCTATGTTCCTTGGG-3'), F7EnvJR (5'-TATCAAAC/TTGGCTGTGGTATATAA-3') and 8 reverse primers; R1EnvJR (5'-CTATCTGTCCCCTCAGCTACTGCTA-3'), R2EnvJR (5'-GCTAAGAATCCATCCACT-AATCGT-3'), R3EnvJR (5'-CCTGCCTAACTCTATTCAC-3'), R4EnvJR (5'-TTCAATT-AG/AGGTGTATATTAAGCCTGTG-3'), R5EnvJR (5'-GCCCCAGACTGTGAGTTGCA-ACAGATG-3'), R6EnvJR (5'-GATGGGAGGGGCATACAT-3'), R7EnvJR (5'-CAGCA-GTTGAGTTGATACTACTGG-3'), R8EnvJR (5'-TTTAGCATCTGATGCACAAAATAG-3') spanning the entire gp160 region and the ABI prism BigDye Terminator sequencing kit (Perkin Elmer) were used in the sequencing reaction. Sequence analysis was performed at the SWEGENE Centre of Genomic Ecology at Lund University. The sequenced segments were assembled to a contig sequence using the ContigExpress of VectorNTI Advance 10 software (Invitrogen). Sequences were aligned using ClustalX [52] followed by manual editing in GeneDoc [53]. For analysis of variation in potentially N-linked glycosylation sites (PNGS) we used the N-glycosite tool in the HIV-1 sequence database [31].

### DC-SIGN binding assay

Ramos/wt and Ramos/DC-SIGN cells, 2 × 10^5 ^diluted in 200 μl of IMDM medium, were pulsed with virus for 3 hours at 37°C. Added virus contained 1 ng/ml of functional reverse transcriptase (RT), as measured by the Cavidi HS Lenti kit. After incubation the cells were washed twice with PBS and lysed with 1% Trition X-100. The concentrations of p24 in cell lysates and added virus were determined by p24 ELISA. Percentage specifically DC-SIGN associated p24 was determined by subtracting Ramos/DC-SIGN associated p24 with Ramos/wt associated p24, dividing with added p24 and multiplying with 100.

### DC-SIGN mediated trans-infection assay

The assay for analysis of DC-SIGN mediated infection was setup according to published protocol [45]. In brief, irradiated Ramos/DC-SIGN cells were suspended in infection medium, i.e. complete medium with 5 U/ml interleukin-2, and seeded into 96-well plates (5 × 10^4 ^cells/well). Ramos/DC-SIGN cells were then pulsed with virus corresponding to 75 pg RT for 3 hours at 37°C. After virus-pulsing the Ramos/DC-SIGN cells were washed twice with PBS, and cocultured with 10^5 ^PHA-activated PBMC or 5 × 10^4 ^C6 cells. As a control for that DC-SIGN is really responsible for the *trans*-infections, the same setup was also done with Ramos/wt. In parallel, for the measure of relative DC-SIGN use efficacy, direct infections of PBMC or C6 cells were setup using the same amount of cells and inoculum virus; however, virus was washed out 24 hours after the infection. Seven days after initiation of *trans*- and direct infections, supernatants were harvested and p24 antigen content was analysed. The relative efficacy of DC-SIGN use was assessed as the ratio of p24 release in DC-SIGN mediated infections over p24 in directly infected cultures.

### Competition assay with primary isolates

In head-to-head competition assays DC-SIGN-mediated and direct PBMC infections were setup as described, with the exception that in each assay two isolates, representing chronic and end-stage R5 viruses, were according to RT activity mixed 1:1. Virus mixes were then added to ten parallel wells and diluted, starting from 75 pg RT/well/isolate, in four or eight fold steps. After seven days supernatants where harvested and p24 antigen content analysed. Cells from p24 positive wells, where virus was diluted to the limit, were chosen for identification of replicating virus using sequencing of the gp120 V2 region. The V2 region was PCR amplified and sequenced as previously described [54] and V2 sequences of R5 isolates (4481, 6322, 1372 and 5714) used in the competition assays were submitted to Genbank and received accession numbers [GenBank: AF417523, AF417526, EF136504 and EF136505].

### Statistical analysis

Non-parametric Wilcoxon's matched pairs test was used in the comparison of DC-SIGN binding and utilization of R5 isolates obtained sequentially before and after AIDS onset at chronic and end-stage disease. Fischer's exact test was used to determine significance when comparing PNGS in chronic and end-stage isolates. Mann-Whitney U-test was used to evaluate the difference between virus isolates having or lacking the V2 and V4 PNGS site in binding and utilization of DC-SIGN. Exact Mann-Whitney test was used for evaluation of competition assays.

## Competing interests

The author(s) declare that they have no competing interests.

## Authors' contributions

MB planned the experiments, carried out cell assays, analyzed sequences and wrote the manuscript. JR generated and sequenced full length *env *clones, analyzed PNGS and helped to draft the manuscript. CK set up and optimized the trans-infection assays. JS, and MJC participated in the *env *cloning. DFJP supplied essential reagents. AK was responsible for the clinical follow up of the patient cohort. JA helped to draft the manuscript. PRG coordinated the env cloning and helped to draft the manuscript. MJ conceived the study, participated in its design, interpreted results and helped to draft the manuscript. All authors read and approved the final manuscript.
